# The Biphasic Role of Microglia in Alzheimer's Disease

**DOI:** 10.1155/2012/737846

**Published:** 2012-05-10

**Authors:** Tetsuya Mizuno

**Affiliations:** Department of Neuroimmunology, Research Institute of Environmental Medicine, Nagoya University, Furo-cho, Chikusa-ku, Nagoya 464-8601, Japan

## Abstract

Neuroinflammation is involved in the pathogenesis of Alzheimer's disease (AD). Microglia, macrophage-like resident immune cells in the brain, play critical roles in the inflammatory aspects of AD. Microglia may be activated by oligomeric and fibrillar species of amyloid **β** (A**β**) that are constituents of senile plaques and by molecules derived from degenerated neurons, such as purines and chemokines, which enhance their migration and phagocytosis. The main neurotoxic molecules produced by activated microglia may be reactive oxygen species, glutamate, and inflammatory cytokines such as tumor-necrosis-factor-**α** and interleukin- (IL-) 1**β** These molecules differentially induce neurotoxicity. A**β** itself directly damages neurons. In terms of neuroprotective properties, microglia treated with fractalkine or IL-34 attenuate A**β** neurotoxicity by A**β** clearance and the production of antioxidants. Therefore, regulation of the microglial role in neuroprotection may be a useful therapeutic strategy for AD.

## 1. Introduction

Microglia, macrophage-like immune cells in the central nervous system (CNS), cluster around the senile plaques that along with polymorphous amyloid *β* (A*β*) deposits are the pathological hallmarks of Alzheimer's disease (AD). Microglia have a biphasic neurotoxic-neuroprotective role in the pathogenesis of AD. In regard to their neurotoxic properties, microglia may be involved in the inflammatory component of AD [1, 2]. In AD, the trigger molecule for microglial activation may be A*β* and molecules derived from degenerated neurons may enhance microglial neurotoxicity [3]. A*β* exists in different assembly forms including monomers, oligomers, and fibrils. Both oligomeric A*β* (oA*β*) and fibrillar A*β* (fA*β*) have been shown to stimulate microglial secretion of proinflammatory cytokines such as interleukin-1 (IL-1), IL-6, and tumor-necrosis-factor-*α* (TNF-*α*); chemokines including monocyte chemotactic-1 (MCP-1) and macrophage inflammatory protein-1 (MIP-1); complement components; free radicals such as reactive oxygen species (ROS), including superoxide anions and hydroxy radicals [4, 5]. Glutamate also plays an important role in microglial neurotoxicity in AD. Activated microglia produce large amounts of glutamate, which induces excitotoxicity via N-methyl-D-aspartate (NMDA) receptor signaling [6–9]. Chronic activation of extrasynaptic NMDA receptors leads to increased A*β* production [10].

**Figure 1:**
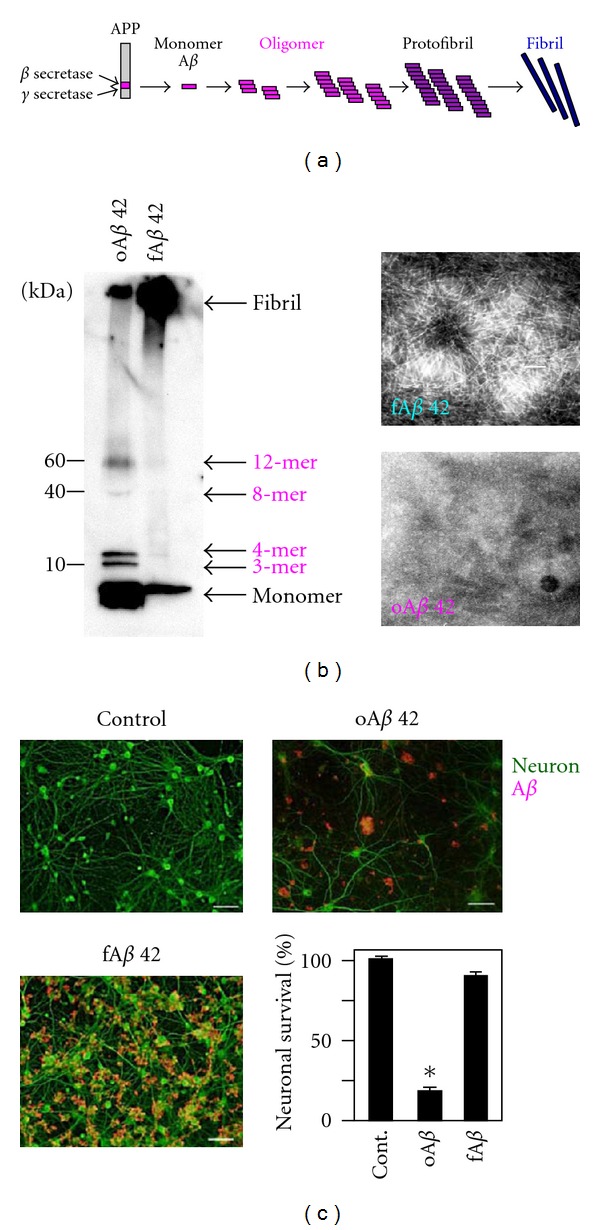
A*β* neurotoxicity. A*β* is derived from APP by enzymatic cleavage. A*β* consists of 40 or 42 amino acids. A*β*40 is soluble and of lower toxicity, but A*β*42 is prone to aggregate and form fibrillar structures via oligomers (a). Oligomeric A*β* (oA*β*42) and fibrillar A*β* (fA*β*42) are detected by western blotting and an electron microscope (b). Administration of 5 *μ*M oA*β*42 to cortical cultures induces significant neuronal death. In contrast, administration of fA*β*42 does not induce neuronal cell death, although A*β* deposition is observed on dendrites (c).

A*β* itself is toxic to neurons in AD, with oA*β* being more toxic than fA*β* (Figure 1). The toxicity of oA*β* manifests itself in terms of synaptic dysfunction, including inhibition of hippocampal long-term potentiation, facilitation of long-term depression, and disruption of synaptic plasticity [11, 12]. It is therefore necessary to evaluate microglial neurotoxicity apart from A*β* neurotoxicity.

In regard to its neuroprotective properties, microglia attenuate A*β* neurotoxicity by A*β* clearance, including phagocytosis and degradation of A*β* and the production of antioxidants and neurotrophic factors [13–15]. In the present paper, we focus on the trigger molecules that mediate microglial activation and the mechanisms of microglial neurotoxicity mediated by ROS glutamate, and inflammatory cytokines. We also discuss the neuroprotective role of microglia in AD.

## 2. The Trigger Molecules That Mediate Microglial Activation

### 2.1. Fibrillar A*β*

A*β*, the key mediator of AD, is processed from the amyloid precursor protein (APP). The most common isoforms are A*β*1-40 and A*β*1-42, which are the major constituents of senile plaques. A*β*1-42 is more prone to aggregate than A*β*1-40. Initial A*β* deposition begins with A*β*1-42, but not with A*β*1-40. In the process of A*β*1-42 aggregation, many types of soluble oA*β* are formed: dimers, trimers, tetramers, dodecamers, A*β*-derived diffusible ligands, and annular protofibrils [16–18]. Stimulation of microglia with fA*β* reportedly results in the Syk kinase- and NF*κ*B-dependent production of TNF-*α*, iNOS, and peroxynitrite [19]. However, the ability of fA*β* to activate microglia is generally low or absent when fA*β* is used as the sole stimulant. Recent reports have clarified that fA*β* can activate microglia via Toll-like receptor 2 (TLR2) [20] or interaction with cell surface receptor complexes. TLR2, TLR4, and TLR6 have been shown to be essential components of the receptor complexes for microglial activation. The coreceptor CD14 and TLR2 and 4 complex is required for fA*β*-stimulated microglial activation [21]. The class B scavenger receptor CD36 and TLR4 and 6 complex is also involved in the activation of microglia by fA*β*. The signals mediated by this receptor complex induce IL-1*β* production in microglia [22].

### 2.2. Oligomeric A*β*

**Figure 2:**
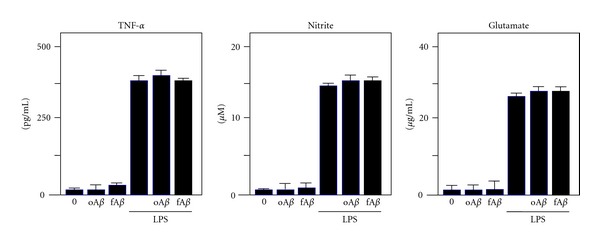
Inflammatory molecules produced by A*β*- or LPS-activated microglia. In primary microglial culture, administration of 5 *μ*M oA*β*42 or 5 *μ*M fA*β*42 for 24 h does not induce the production of neurotoxic mediators such as TNF-*α*, glutamate, or nitrite, a stable breakdown product of NO. While microglia activated with 1 *μ*g/mL LPS produce these molecules, both oA*β*42 and fA*β*42 do not enhance the production.

The patterns of microglial activation caused by fA*β* and oA*β* are different. In addition, studies of microglial activation with oA*β* have yielded controversial results. oA*β* activates microglia by increasing levels of phosphorylated Lyn, Syk kinase, and p38 MAP kinase, which results in the production of IL-6 and a decrease in MCP-1 [23]. On the other hand, oA*β* does not produce several proinflammatory mediators commonly induced by lipopolysaccharides (LPS), such as prostaglandin E2, glutamate, TNF-*α*, IL-1*β*, and IL-6. There is a report that oA*β* at low nanomolar concentrations induces neurotoxicity by increasing the production of nitric oxide (NO) as well as the activity of scavenger receptor A and the Ca^2+^-activated potassium channel KCa3.1 [24]. oA*β* induces neuronal ROS through a mechanism requiring NMDA receptor activation [25]. ROS is also induced by fA*β* and oA*β* in microglia [26]. In contrast to the above reports, our data showed that both fA*β* and oA*β* failed to induce toxic molecules such as TNF-*α*, NO, and glutamate in microglia and to enhance these molecules in LPS-activated microglia (Figure 2) [15]. The synthetic oA*β* that is used for in vitro studies contains oligomers of different sizes and shapes, and microglia may respond to this heterogeneous oA*β* mixture in various ways. Moreover, synthetic oA*β* preparations are less potent than oA*β* isolated from the supernatant of transfected cell cultures.

### 2.3. The Molecules Derived from Degenerated Neurons

Recent findings have indicated that damaged neurons are not merely passive targets of microglia but rather regulate microglial activity through nucleotides and chemokines [27]. Damaged neurons release several substances that activate microglia, for instance purines, including ATP [28] and UDP [29]; chemokines, such as CCL-21 [30]; glutamate [31]. However, these molecules do not always induce microglial neurotoxicity. ATP regulates microglial branch dynamics and mediates a rapid microglial response toward injured neurons. UDP triggers microglial phagocytosis via P2Y6 receptors. The chemokine CCL-21, released by damaged neurons, activates microglia via the CXCR3 receptor. Excessive neuronal glutamate is released as a result of neurodegenerative processes. Microglial cells express the AMPA and kainate subtypes of ionotropic glutamate receptors [32]. Metabotropic glutamate (mGlu) receptor 2, mGlu3, and mGlu5 are expressed in microglia [33, 34]. Glutamate serves as an activation signal for microglia, and activation of mGluR2 on microglia promotes neurotoxicity. However, microglial mGluR5 provides neuronal protection by suppressing the NO and TNF-*α* production induced by blood protein fibrinogen [34].

## 3. The Neurotoxic Molecules Produced by Microglia

### 3.1. ROS

Oxidative damage to protein, lipids, polysaccharides, and DNA is involved in the pathogenesis of AD [35]. The expression of NADPH oxidase, a multisubunit enzyme complex responsible for the production of ROS, is upregulated in AD [36]. Microglial NADPH oxidase is activated by fA*β* [37, 38], and NADPH oxidase activation subsequently causes neurotoxicity through two mechanisms: (1) extracellular ROS produced by microglia are directly toxic to neurons, and (2) intracellular ROS function as a signaling mechanism in microglia to amplify the production of several proinflammatory and neurotoxic cytokines [39]. ROS are induced in the mitochondrial membranes of both neurons and microglia, causing subsequent oxidative damage in the early stages of disease progression. Loss of mitochondrial membrane potential and increase of ROS production have been demonstrated in studies of AD patients as well as in transgenic mice models of AD [40, 41]. An increase in hydrogen peroxide and a decrease in cytochrome oxidase activity were found in young Tg2576 mice prior to the appearance of A*β* plaques [40]. Oxidative stress has been shown to contribute to the onset of cognitive dysfunction caused by A*β* [42].

A recent report showed that fA*β* and oA*β* induced ROS in microglia through the TRPV1 cation channel, also known as the capsaisin receptor, and that pretreatment with fA*β* or oA*β* induced microglial priming through Kv1.3 K(+) channels, that is, increased ROS production upon secondary stimulation with the phorbol ester PMA [26]. The translocation of chloride intracellular channel 1 from the cytosol to the plasma membrane is also involved in microglial ROS generation [43].

Neuronal ROS induced by A*β* can be selectively dysfunctional as well as degenerative. Overstimulation of excitatory NMDA receptors can lead to excessive ROS. Antioxidative enzyme heme oxygenase-1 (HO-1) is a microsomal enzyme that oxidatively cleaves heme to produce biliverdin, carbon monoxide, and iron [44]. A*β* binds to heme to promote a functional heme deficiency and induces mitochondrial dysfunction and neurotoxicity [45]. APP also binds to HO, and oxidative neurotoxicity is markedly enhanced in cerebral cortical cultures from APP Swedish mutant transgenic mice [46].

### 3.2. Glutamate

Activated microglia release large amounts of glutamate through upregulation of glutaminase expression and induce excitoneurotoxicity through NMDA receptor signaling [7, 47, 48]. Microglial glutamate production is reported to be induced by APP, but not A*β* [48]. Excessive glutamate increases Ca^2+^ influx via the NMDA receptor, resulting in Ca^2+^/calmodulin-dependent protein kinase (CaMK) activation. NO induced by CaMK inhibits mitochondrial function. Stimulation of synaptic NMDA receptors enhances prosurvival signals through the activation of cAMP response element-binding protein (CREB) and the extracellular signal-regulated kinase (ERK) cascade [49, 50], whereas calcium flux through extrasynaptic NMDA receptors overrides these functions, causing mitochondrial dysfunction and neuronal cell death [51, 52]. A recent report suggested that chronic activation of extrasynaptic NMDA receptors leads to sustained neuronal A*β* release via amyloidogenic APP expression [53].

Focal bead-like swelling in dendrites and axons, known as neuritic beading, is a neuropathological sign that is a feature of neuronal cell dysfunction preceding neuronal death in various diseases such as ischemia, epilepsy, brain tumor, and AD [54–57]. We found that glutamate from activated microglia induces neuritic beading by impairing dendritic and axonal transport through NMDA receptor signaling [7]. Moreover, we demonstrated that gap junction hemichannels are the main avenue of excessive glutamate release from neurotoxic activated microglia [6]. The blockade of gap junction hemichannels by glycyrrhetinic acid derivatives significantly prevents activated microglia-mediated neuronal death in vitro [7, 58, 59] and in vivo in rodent models of transient ischemic brain injury, multiple sclerosis, amyotrophic lateral sclerosis, and AD [60–62]. In the APP/PS1 transgenic mouse model of AD, glycyrrhetinic acid derivatives improved memory impairments without altering A*β* deposition [62].

oA*β* is directly neurotoxic as a result of inducing glutamate release from hippocampal neurons and may contribute to dysregulation of excitatory signaling in neurons [63].

### 3.3. Inflammatory Cytokines

oA*β*, but not fA*β*, has been shown to increase levels of TNF-*α* and IL-1*β* in rat microglial cultures [64]. However, gene expression analysis of microglia using cDNA arrays has confirmed that the upregulation of TNF-*α* and IL-1*β* is caused by both oA*β* and fA*β* [65]. TNF-*α* is a well-characterized proinflammatory cytokine involved in many neuroinflammatory cascades, including autocrine activation of microglia [66] and direct apoptosis via activation of extrinsic pathway-associated TNF receptors [67, 68]. TNF-*α* enhances microglial glutaminase expression, glutamate production, and cell-surface expression of gap junction hemichannels [6]. Synergistic and autocrine activities of TNF-*α* may cause the release of large amounts of glutamate, resulting in excitotoxic neuronal death [69]. TNF-*α* has been shown to directly upregulate the expression of the AMPA receptor GluR1 subunit in mouse hippocampus and cerebral cortex neurons [70] and to exacerbate AMPA-induced neuronal death at high doses [71]. TNF-*α* has also been reported to enhance excitotoxicity through synergistic stimulation of the TNF and NMDA receptors [72].

IL-1*β* is known to be a driving force in the inflammatory process in AD, and it promotes the synthesis and processing of APP [73]. IL-1*β* affects ion currents, intracellular Ca^2+^ homeostasis, and membrane potentials and suppresses long-term potentiation, thus contributing to dysfunction and inflammation [74]. A recent report indicated that the cytoplasmic receptor NALP3 inflammasome is involved in the innate immune response in AD [75]. Activation of the microglial NALP3 inflammasome is initiated by phagocytosis of fA*β* and induces lysosomal damage and cathepsin B release. Moreover, it leads to the cleavage of pro-IL-1*β*/pro-IL-18 into IL-1*β*/IL-18 by caspase-1. Subsequently, IL-1*β* activates the secretion of several proinflammatory and chemotactic mediators [75].

Wnt signaling plays an important role in neural development, including synaptic differentiation. Wnt 5a and its receptor Frizzled-5 have been shown to be upregulated in the AD mouse brain [76], and activation of Wnt5a signaling enhances A*β*-evoked neurotoxicity by induced TNF-*α* and IL-1*β* [76]. In contrast, postsynaptic damage induced by oA*β* in hippocampal neurons is reported to be prevented by Wnt5a [77]. This discrepancy can be explained by the fact that basal Wnt5a has synaptoprotective activity, but excessive Wnt5a may induce proinflammatory factors.

## 4. Phagocytosis of Microglia

Microglial phagocytosis of neuronal debris and A*β* plays a pivotal role in AD. Phagocytosis is associated with inflammation during uptake of microbes via TLRs and Fc receptors, while phagocytosis of apoptotic cells is executed without inflammation via phosphatidylserine receptors such as T-cell-immunoglobulin-mucin-4 (TIM-4) [78, 79]. Milk-fat-globule-EGF-factor-8 (MFG-E8), secreted by activated microglia or macrophages, also binds to phosphatidylserine exposed on plasma membranes of apoptotic cells [80, 81]. Phagocytosis with inflammation may be toxic to neurons because of the production of inflammatory molecules such as proinflammatory cytokines, NO, and ROS. However, phagocytosis of A*β* contributes to microglial neuroprotection in AD. Peptidoglycan, the TLR2 ligand, and unmethylated DNA CpG motifs, the TLR9 ligand, increase A*β* phagocytosis through protein-coupled formyl peptide receptor-like 2 [82, 83]. Similarly, LPS, the TLR4 ligand, increases phagocytosis through the CD14 receptor [84]. TLR4 mutation exacerbates the A*β* burden in mouse models of AD [85].

## 5. Molecules Able to Induce Microglial Neuroprotective Properties

### 5.1. Fractalkine

Degenerating neurons produce signaling molecules that regulate microglial phagocytosis and neuroprotection. Some of this signaling may be controlled by chemokines and chemokine receptors, which are widely expressed throughout the central nervous system [86]. We have shown that the soluble CX3C chemokine fractalkine (FKN), secreted from damaged neurons, promotes microglial phagocytosis of neuronal debris through the release of MFG-E8 and induces the expression of the antioxidant enzyme HO-1 in microglia, resulting in neuroprotection against glutamate toxicity [87]. The end-products of HO-1 including biliverdin, carbon monoxide, and iron provide cellular and tissue protection through antiinflammatory, antiapoptotic, or antioxidative effects [88]. Numerous studies have demonstrated that upregulation of HO-1 expression in the CNS may be beneficial to counteract neuroinflammation and neurodegenerative diseases [89]. The neuroprotective effect of FKN is abolished by treatment with the HO-1 inhibitor tin-mesoporphyrin IX (SnMP). Moreover, FKN suppresses microglial NO, IL-6, and TNF-*α* production [90]. FKN signaling is deficient in AD brains and is downregulated by A*β*. CX3CR1, the fractalkine receptor, is a key member of the microglial pathway that protects against AD-related cognitive deficits that are associated with aberrant microglial activation and elevated inflammatory cytokines [91]. Mice lacking the CX3CR1 receptor show cognitive dysfunction as demonstrated by contextual fear conditioning and Morris water maze tests, deficits in motor learning, and a significant impairment in long-term potentiation via increase in IL-1*β* [92]. CX3CR1 deficiency worsens the AD-related neuronal and behavioral deficits [91]. In contrast, CX3CR1 deficiency is reported to reduce A*β* deposition in AD mouse models [93]. Thus, FKN-CX3CR1 signaling in AD is still controversial.

### 5.2. IL-34

The dimeric glycoprotein IL-34, which is mainly expressed in neurons, may also be a neuronal cytokine that regulates microglial function. The major function of IL-34 is to stimulate the differentiation and proliferation of monocytes and macrophages via the colony-stimulating-factor- (CSF-) 1 receptor [94]. We have shown that IL-34 induces microglial proliferation and antioxidant HO-1 production and enhances the degradation of A*β* via insulin degrading enzyme (IDE) (also known as A*β* degrading enzyme) and that IL-34 reduces the amount of oA*β* and ROS present in the supernatant of neuron-microglia cocultures, resulting in microglial neuroprotection against oA*β* toxicity [95]. IDE activity is critical in determining the level of A*β*. The levels of hippocampal IDE protein and activity have been shown to be reduced in AD [96]. Enhanced IDE activity in IDE/APP double-transgenic mice decreased A*β* levels and prevented the development of AD pathology [97]. Moreover, single intracerebroventricular injection of IL-34 effectively suppressed the impairment of associative learning in an APP/PS1 transgenic mouse model of AD [95]. The injection of IL-34 may act directly on microglia, which can rapidly eliminate oA*β* via upregulation of IDE and exert antioxidant effect via HO-1.

### 5.3. M-CSF

CSF-1, also known as macrophage colony-stimulating factor (M-CSF), is another ligand of the CSF-1 receptor. M-CSF enables the acidification of macrophage lysosomes and subsequently the degradation of internalized A*β* [98]. Intraperitoneal injection of M-CSF prevents memory disturbance in APP/PS1 mice by inducing microglial phagocytosis of A*β* [99]. A recent report showed that IL-34 and M-CSF differ in their structure and the CSF-1 receptor domains that they bind, causing different bioactivities and signal activation kinetics [100]. Both IL-34 and M-CSF are useful molecules in terms of inducing microglial neuroprotective properties.

### 5.4. CpG

TLR9, which is located in the intracellular endosomal-lysosomal compartment in innate immune cells, detects single-stranded DNA containing unmethylated CpG. Microglia express TLR9 at higher levels than do astrocytes and neuronal cells. Thus, CpG mainly acts on microglia in the CNS. We have also shown that microglia activated by CpG attenuate oA*β* neurotoxicity [15]. While high concentrations of CpG (10 *μ*M) induce TNF-*α*, IL-12, and NO in microglia and enhance neuronal damage [101, 102], lower concentrations of CpG (1 nM–100 nM) enhance microglial phagocytosis of A*β* without inflammation. Intracerebroventricular administration of CpG ameliorates both the cognitive impairments induced by oA*β* and the impairment of associative learning in a Tg2576 mouse model of AD [15].

Taken together, these molecules induce neuroprotective properties in microglia through the antioxidant effect of HO-1 and A*β* clearance. HO-1 is induced by nuclear translocation of Nrf2, which is a transcription factor and reportedly plays a pivotal role in cellular survival [103, 104]. Nrf2 gene therapy also has been shown to improve memory in the mouse model of AD [105]. Moreover, A*β* clearance including phagocytosis and degradation of A*β* by microglia can decrease A*β* plaque formation and A*β* toxicity.

## 6. Conclusion

**Figure 3:**
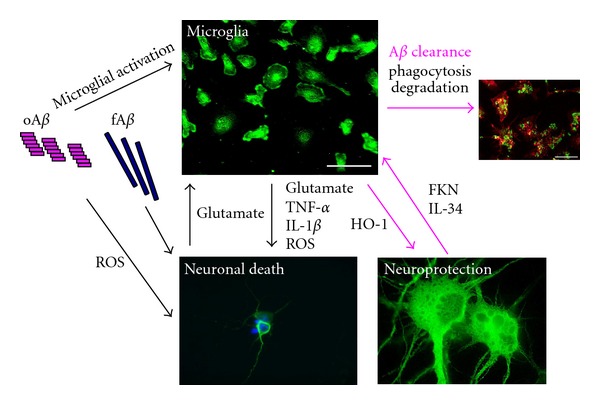
A*β* toxicity in neurons and microglia. A*β* is directly toxic to neurons by ROS, and enhances the production of microglia-derived neurotoxic molecules such as glutamate, TNF-*α*, and IL-1*β*. Glutamate from degenerated neurons also induces microglial neurotoxicity. IL-34 and FKN induce microglial neuroprotection via A*β* clearance and antioxidant activity.

While A*β*, especially oA*β*, is directly toxic to neurons, it may also enhance microglial neurotoxic effects by inducing inflammatory mediators. Degenerated neurons produce molecules other than A*β* that enhance microglial neurotoxicity. However, the microglial neuroprotective effect resulting from A*β* clearance and antioxidant activity is obvious in AD (Figure 3). The conditions that determine microglial toxic or protective effects remain to be elucidated. Clarifying these issues may contribute to the understanding of AD pathophysiology. A useful therapeutic strategy for AD may be to regulate microglia toward neuroprotection, specifically, A*β* clearance without inflammation.

## References

[1] Meda L, Cassatella MA, Szendrei GI (1995). Activation of microglial cells by *β*-amyloid protein and interferon-*γ*. *Nature*.

[2] Akiyama H, Barger S, Barnum S (2000). Key issues in Alzheimer’s disease inflammation. *Neurobiology of Aging*.

[3] Pais TF, Figueiredo C, Peixoto R, Braz MH, Chatterjee S (2008). Necrotic neurons enhance microglial neurotoxicity through induction of glutaminase by a MyD88-dependent pathway. *Journal of Neuroinflammation*.

[4] McGeer PL, McGeer EG (2001). Inflammation, autotoxicity and Alzheimer disease. *Neurobiology of Aging*.

[5] Della Bianca V, Dusi S, Bianchini E, Dal Prà I, Rossi F (1999). *β*-amyloid activates the O_2_forming NADPH oxidase in microglia, monocytes, and neutrophils. A possible inflammatory mechanism of neuronal damage in Alzheimer’s disease. *Journal of Biological Chemistry*.

[6] Takeuchi H, Jin S, Wang J (2006). Tumor necrosis factor-*α* induces neurotoxicity via glutamate release from hemichannels of activated microglia in an autocrine manner. *Journal of Biological Chemistry*.

[7] Takeuchi H, Mizuno T, Zhang G (2005). Neuritic beading induced by activated microglia is an early feature of neuronal dysfunction toward neuronal death by inhibition of mitochondrial respiration and axonal transport. *Journal of Biological Chemistry*.

[8] Barger SW, Basile AS (2001). Activation of microglia by secreted amyloid precursor protein evokes release of glutamate by cystine exchange and attenuates synaptic function. *Journal of Neurochemistry*.

[9] Piani D, Spranger M, Frei K, Schaffner A, Fontana A (1992). Macrophage-induced cytotoxicity of N-methyl-D-aspartate receptor positive neurons involves excitatory amino acids rather than reactive oxygen intermediates and cytokines. *European Journal of Immunology*.

[10] Bordji K, Becerril-Ortega J, Nicole O, Buisson A (2010). Activation of extrasynaptic, but not synaptic, NMDA receptors modifies amyloid precursor protein expression pattern and increases amyloid-*β* production. *Journal of Neuroscience*.

[11] Walsh DM, Klyubin I, Fadeeva JV (2002). Naturally secreted oligomers of amyloid *β* protein potently inhibit hippocampal long-term potentiation *in vivo*. *Nature*.

[12] Li S, Hong S, Shepardson NE, Walsh DM, Shankar GM, Selkoe D (2009). Soluble oligomers of amyloid *β* protein facilitate hippocampal long-term depression by disrupting neuronal glutamate uptake. *Neuron*.

[13] Yan P, Hu X, Song H (2006). Matrix metalloproteinase-9 degrades amyloid-*β* fibrils *in vitro* and compact plaques *in situ*. *Journal of Biological Chemistry*.

[14] Richard KL, Filali M, Préfontaine P, Rivest S (2008). Toll-like receptor 2 acts as a natural innate immune receptor to clear amyloid *β*1-42 and delay the cognitive decline in a mouse model of Alzheimer’s disease. *Journal of Neuroscience*.

[15] Doi Y, Mizuno T, Maki Y (2009). Microglia activated with the toll-like receptor 9 ligand CpG attenuate oligomeric amyloid *β* neurotoxicity in *in vitro* and *in vivo* models of Alzheimer’s disease. *American Journal of Pathology*.

[16] Shankar GM, Li S, Mehta TH (2008). Amyloid-*β* protein dimers isolated directly from Alzheimer’s brains impair synaptic plasticity and memory. *Nature Medicine*.

[17] Lesné S, Ming TK, Kotilinek L (2006). A specific amyloid-*β* protein assembly in the brain impairs memory. *Nature*.

[18] Kayed R, Pensalfini A, Margol L (2009). Annular protofibrils area structurally and functionally distinct type of amyloid oligomer. *Journal of Biological Chemistry*.

[19] Combs CK, Colleen Karlo J, Kao SC, Landreth GE (2001). *β*-amyloid stimulation of microglia anti monocytes results in TNF *α*-dependent expression of inducible nitric oxide synthase and neuronal apoptosis. *Journal of Neuroscience*.

[20] Jana M, Palencia CA, Pahan K (2008). Fibrillar amyloid-*β* peptides activate microglia via TLR2: Implications for Alzheimer’s disease. *Journal of Immunology*.

[21] Reed-Geaghan EG, Savage JC, Hise AG, Landreth GE (2009). CD14 and toll-like receptors 2 and 4 are required for fibrillar A*β*-stimulated microglial activation. *Journal of Neuroscience*.

[22] Stewart CR, Stuart LM, Wilkinson K (2010). CD36 ligands promote sterile inflammation through assembly of a Toll-like receptor 4 and 6 heterodimer. *Nature Immunology*.

[23] Sondag CM, Dhawan G, Combs CK (2009). *β* amyloid oligomers and fibrils stimulate differential activation of primary microglia. *Journal of Neuroinflammation*.

[24] Maezawa I, Zimin PI, Wulff H, Jin LW (2011). Amyloid-*β* protein oligomer at low nanomolar concentrations activates microglia and induces microglial neurotoxicity. *Journal of Biological Chemistry*.

[25] de Felice FG, Velasco PT, Lambert MP (2007). A*β* oligomers induce neuronal oxidative stress through an N-methyl-D-aspartate receptor-dependent mechanism that is blocked by the Alzheimer drug memantine. *Journal of Biological Chemistry*.

[26] Schilling T, Eder C (2011). Amyloid-*β*-induced reactive oxygen species production and priming are differentially regulated by ion channels in microglia. *Journal of Cellular Physiology*.

[27] Biber K, Neumann H, Inoue K, Boddeke HWGM (2007). Neuronal ‘On’ and ‘Off’ signals control microglia. *Trends in Neurosciences*.

[28] Davalos D, Grutzendler J, Yang G (2005). ATP mediates rapid microglial response to local brain injury *in vivo*. *Nature Neuroscience*.

[29] Koizumi S, Shigemoto-Mogami Y, Nasu-Tada K (2007). UDP acting at P2Y6 receptors is a mediator of microglial phagocytosis. *Nature*.

[30] de Jong EK, Dijkstra IM, Hensens M (2005). Vesicle-mediated transport and release of CCL21 in endangered neurons: a possible explanation for microglia activation remote from a primary lesion. *Journal of Neuroscience*.

[31] Färber K, Kettenmann H (2005). Physiology of microglial cells. *Brain Research Reviews*.

[32] Noda M, Nakanishi H, Nabekura J, Akaike N (2000). AMPA-kainate subtypes of glutamate receptor in rat cerebral microglia. *Journal of Neuroscience*.

[33] Taylor DL, Diemel LT, Cuzner ML, Pocock JM (2002). Activation of group II metabotropic glutamate receptors underlies microglial reactivity and neurotoxicity following stimulation with chromogranin A, a peptide up-regulated in Alzheimer’s disease. *Journal of Neurochemistry*.

[34] Piers TM, Heales SJ, Pocock JM (2011). Positive allosteric modulation of metabotropic glutamate receptor 5 down-regulates fibrinogen-activated microglia providing neuronal protection. *Neuroscience Letters*.

[35] Lyras L, Cairns NJ, Jenner A, Jenner P, Halliwell B (1997). An assessment of oxidative damage to proteins, lipids, and DNA in brain from patients with Alzheimer’s disease. *Journal of Neurochemistry*.

[36] Shimohama S, Tanino H, Kawakami N (2000). Activation of NADPH oxidase in Alzheimer’s disease brains. *Biochemical and Biophysical Research Communications*.

[37] El Khoury J, Hickman SE, Thomas CA, Cao L, Silverstein SC, Loike JD (1996). Scavenger receptor-mediated adhesion of microglia to *β*-amyloid fibrils. *Nature*.

[38] McDonald DR, Brunden KR, Landreth GE (1997). Amyloid fibrils activate tyrosine kinase-dependent signaling and superoxide production in microglia. *Journal of Neuroscience*.

[39] Block ML (2008). NADPH oxidase as a therapeutic target in Alzheimer’s disease. *BMC Neuroscience*.

[40] Manczak M, Anekonda TS, Henson E, Park BS, Quinn J, Reddy PH (2006). Mitochondria are a direct site of A*β* accumulation in Alzheimer’s disease neurons: Implications for free radical generation and oxidative damage in disease progression. *Human Molecular Genetics*.

[41] Reddy PH, Beal MF (2008). Amyloid *β*, mitochondrial dysfunction and synaptic damage: implications for cognitive decline in aging and Alzheimer’s disease. *Trends in Molecular Medicine*.

[42] Alkam T, Nitta A, Mizoguchi H, Itoh A, Nabeshima T (2007). A natural scavenger of peroxynitrites, rosmarinic acid, protects against impairment of memory induced by A*β*25-35. *Behavioural Brain Research*.

[43] Milton RH, Abeti R, Averaimo S (2008). CLIC1 function is required for *β*-amyloid-induced generation of reactive oxygen species by microglia. *Journal of Neuroscience*.

[44] Maines MD (1997). The heme oxygenase system: a regulator of second messenger gases. *Annual Review of Pharmacology and Toxicology*.

[45] Atamna H, Frey WH (2004). A role for heme in Alzheimer’s disease: heme binds amyloid *β* and has altered metabolism. *Proceedings of the National Academy of Sciences of the United States of America*.

[46] Takahashi M, Doré S, Ferris CD (2000). Amyloid precursor proteins inhibit heme oxygenase activity and augment neurotoxicity in Alzheimer’s disease. *Neuron*.

[47] Piani D, Spranger M, Frei K, Schaffner A, Fontana A (1992). Macrophage-induced cytotoxicity of N-methyl-D-aspartate receptor positive neurons involves excitatory amino acids rather than reactive oxygen intermediates and cytokines. *European Journal of Immunology*.

[48] Barger SW, Basile AS (2001). Activation of microglia by secreted amyloid precursor protein evokes release of glutamate by cystine exchange and attenuates synaptic function. *Journal of Neurochemistry*.

[49] Hardingham GE, Fukunaga Y, Bading H (2002). Extrasynaptic NMDARs oppose synaptic NMDARs by triggering CREB shut-off and cell death pathways. *Nature Neuroscience*.

[50] Ivanov A, Pellegrino C, Rama S (2006). Opposing role of synaptic and extrasynaptic NMDA receptors in regulation of the extracellular signal-regulated kinases (ERK) activity incultured rat hippocampal neurons. *Journal of Physiolgy*.

[51] le veille F, El Gaamouch F, Gouix E (2008). Neuronal viability is controlled by a functional relation between synaptic and extrasynaptic NMDA receptors. *The FASEB Journal*.

[52] Stanika RI, Pivovarova NB, Brantner CA, Watts CA, Winters CA, Andrews SB (2009). Coupling diverse routes of calcium entry to mitochondrial dysfunction and glutamate excitotoxicity. *Proceedings of the National Academy of Sciences of the United States of America*.

[53] Bordji K, Becerril-Ortega J, Nicole O, Buisson A (2010). Activation of extrasynaptic, but not synaptic, NMDA receptors modifies amyloid precursor protein expression pattern and increases amyloid-*β* production. *Journal of Neuroscience*.

[54] Hori N, Carpenter DO (1994). Functional and morphological changes induced by transient *in vivo* ischemia. *Experimental Neurology*.

[55] Swann JW, Al-Noori S, Jiang M, Lee CL (2000). Spine loss and other dendritic abnormalities in epilepsy. *Hippocampus*.

[56] Goel S, Wharton SB, Brett LP, Whittle IR (2003). Morphological changes and stress responses in neurons in cerebral cortex infiltrated by diffuse astrocytoma. *Neuropathology*.

[57] Dickson TC, King CE, McCormack GH, Vickers JC (1999). Neurochemical diversity of dystrophic neurites in the early and late stages of Alzheimer’s disease. *Experimental Neurology*.

[58] Liang J, Takeuchi H, Doi Y (2008). Excitatory amino acid transporter expression by astrocytes is neuroprotective against microglial excitotoxicity. *Brain Research*.

[59] Yawata I, Takeuchi H, Doi Y, Liang J, Mizuno T, Suzumura A (2008). Macrophage-induced neurotoxicity is mediated by glutamate and attenuated by glutaminase inhibitors and gap junction inhibitors. *Life Sciences*.

[60] Takeuchi H, Jin S, Suzuki H (2008). Blockade of microglial glutamate release protects against ischemic brain injury. *Experimental Neurology*.

[61] Shijie J, Takeuchi H, Yawata I (2009). Blockade of glutamate release from microglia attenuates experimental autoimmune encephalomyelitis in mice. *Tohoku Journal of Experimental Medicine*.

[62] Takeuchi H, Mizoguchi H, Doi Y (2011). Blockade of gap junction hemichannel suppresses disease progression in mouse models of amyotrophic lateral sclerosis and Alzheimer’s disease. *PLoS One*.

[63] Brito-Moreira J, Paula-Lima AC, Bomfim TR (2011). A*β* oligomers induce glutamate release from hippocampal neurons. *Current Alzheimer Research*.

[64] Jiao J, Xue B, Zhang L (2008). Triptolide inhibits amyloid-*β*1-42-induced TNF-*α* and IL-1*β* production in cultured rat microglia. *Journal of Neuroimmunology*.

[65] Sebastiani G, Morissette C, Lagacé C (2006). The cAMP-specific phosphodiesterase 4B mediates A*β*-induced microglial activation. *Neurobiology of Aging*.

[66] Kuno R, Wang J, Kawanokuchi J, Takeuchi H, Mizuno T, Suzumura A (2005). Autocrine activation of microglia by tumor necrosis factor-*α*. *Journal of Neuroimmunology*.

[67] Kaul M, Garden GA, Lipton SA (2001). Pathways to neuronal injury and apoptosis in HIV-associated dementia. *Nature*.

[68] Taylor DL, Jones F, Chen Seho Kubota ESF, Pocock JM (2005). Stimulation of microglial metabotropic glutamate receptor mGlu2 triggers tumor necrosis factor *α*-induced neurotoxicity in concert with microglial-derived Fas ligand. *Journal of Neuroscience*.

[69] Eugenín EA, Eckardt D, Theis M, Willecke K, Bennett MVL, Sáez JC (2001). Microglia at brain stab wounds express connexin 43 and *in vitro* form functional gap junctions after treatment with interferon-*γ* and tumor necrosis factor-*α*. *Proceedings of the National Academy of Sciences of the United States of America*.

[70] Yu Z, Cheng G, Wen X, Wu GD, Lee WT, Pleasure D (2002). Tumor necrosis factor *α* increases neuronal vulnerability to excitotoxic necrosis by inducing expression of the AMPA-glutamate receptor subunit GluR1 via an acid sphingomyelinase-and NF-*κ*B-dependent mechanism. *Neurobiology of Disease*.

[71] Bernardino L, Xapelli S, Silva AP (2005). Modulator effects of interleukin-1*β* and tumor necrosis factor-*α* on AMPA-induced excitotoxicity in mouse organotypic hippocampal slice cultures. *Journal of Neuroscience*.

[72] Floden AM, Li S, Combs CK (2005). *β*-Amyloid-stimulated microglia induce neuron death via synergistic stimulation of tumor necrosis factor *α* and NMDA receptors. *Journal of Neuroscience*.

[73] Moore AH, O’Banion MK (2002). Neuroinflammation and anti-inflammatory therapy for Alzheimer’s disease. *Advanced Drug Delivery Reviews*.

[74] Lynch MA (1998). Age-related impairment in long-term potentiation in hippocampus: A role for the cytokine, interleukin-1*β*?. *Progress in Neurobiology*.

[75] Halle A, Hornung V, Petzold GC (2008). The NALP3 inflammasome is involved in the innate immune response to amyloid-*β*. *Nature Immunology*.

[76] Torres E, Gutierrez-Lopez MD, Mayado A, Rubio A, O’Shea E, Colado MI (2011). Changes in interleukin-1 signal modulators induced by 3,4-methylenedioxymethamphetamine (MDMA): regulation by CB2 receptors and implications for neurotoxicity. *Journal of Neuroinflammation*.

[77] Cerpa W, Farías GG, Godoy JA, Fuenzalida M, Bonansco C, Inestrosa NC (2010). Wnt-5a occludes A*β* oligomer-induced depression of glutamatergic transmission in hippocampal neurons. *Molecular Neurodegeneration*.

[78] Neumann H, Kotter MR, Franklin RJM (2009). Debris clearance by microglia: an essential link between degeneration and regeneration. *Brain*.

[79] Miyanishi M, Tada K, Koike M, Uchiyama Y, Kitamura T, Nagata S (2007). Identification of Tim4 as a phosphatidylserine receptor. *Nature*.

[80] Hanayama R, Tanaka M, Miwa K, Shinohara A, Iwamatsu A, Nagata S (2002). Identification of a factor that links apoptotic cells to phagocytes. *Nature*.

[81] Leonardi-Essmann F, Emig M, Kitamura Y, Spanagel R, Gebicke-Haerter PJ (2005). Fractalkine-upregulated milk-fat globule EGF factor-8 protein in cultured rat microglia. *Journal of Neuroimmunology*.

[82] Iribarren P, Chen K, Hu J (2005). CpG-containing oligodeoxynucleotide promotes microglial cell uptake of amyloid *β* 1-42 peptide by up-regulating the expression of the G-protein-coupled receptor mFPR2. *The FASEB Journal*.

[83] Chen K, Iribarren P, Hu J (2006). Activation of toll-like receptor 2 on microglia promotes cell uptake of Alzheimer disease-associated amyloid *β* peptide. *Journal of Biological Chemistry*.

[84] Liu Y, Walter S, Stagi M (2005). LPS receptor (CD14): a receptor for phagocytosis of Alzheimer’s amyloid peptide. *Brain*.

[85] Tahara K, Kim HD, Jin JJ, Maxwell JA, Li L, Fukuchi KI (2006). Role of toll-like receptor signalling in A*β* uptake and clearance. *Brain*.

[86] Tran PB, Miller RJ (2003). Chemokine receptors: signposts to brain development and disease. *Nature Reviews Neuroscience*.

[87] Noda M, Doi Y, Liang J (2011). Fractalkine attenuates excito-neurotoxicity via microglial clearance of damaged neurons and antioxidant enzyme heme oxygenase-1 expression. *Journal of Biological Chemistry*.

[88] Morse D, Lin L, Choi AMK, Ryter SW (2009). Heme oxygenase-1, a critical arbitrator of cell death pathways in lung injury and disease. *Free Radical Biology and Medicine*.

[89] Syapin PJ (2008). Regulation of haeme oxygenase-1 for treatment of neuroinflammation and brain disorders. *British Journal of Pharmacology*.

[90] Mizuno T, Kawanokuchi J, Numata K, Suzumura A (2003). Production and neuroprotective functions of fractalkine in the central nervous system. *Brain Research*.

[91] Cho SH, Sun B, Zhou Y (2011). CX3CR1 protein signaling modulates microglial activation and protects against plaque-independent cognitive deficits in a mouse model of Alzheimer disease. *Journal of Biological Chemistry*.

[92] Rogers JT, Morganti JM, Bachstetter AD (2011). CX3CR1 deficiency leads to impairment of hippocampal cognitive function and synaptic plasticity. *Journal of Neuroscience*.

[93] Lee S, Varvel NH, Konerth ME (2010). CX3CR1 deficiency alters microglial activation and reduces *β*-amyloid deposition in two Alzheimer’s disease mouse models. *American Journal of Pathology*.

[94] Lin H, Lee E, Hestir K (2008). Discovery of a cytokine and its receptor by functional screening of the extracellular proteome. *Science*.

[95] Mizuno T, Doi Y, Mizoguchi H (2011). Interleukin-34 selectively enhances the neuroprotective effects of microglia to attenuate oligomeric amyloid-*β* neurotoxicity. *The American Journal of Pathology*.

[96] Zhao Z, Xiang Z, Haroutunian V, Buxbaum JD, Stetka B, Pasinetti GM (2007). Insulin degrading enzyme activity selectively decreases in the hippocampal formation of cases at high risk to develop Alzheimer’s disease. *Neurobiology of Aging*.

[97] Leissring MA, Farris W, Chang AY (2003). Enhanced proteolysis of *β*-amyloid in APP transgenic mice prevents plaque formation, secondary pathology, and premature death. *Neuron*.

[98] Majumdar A, Cruz D, Asamoah N (2007). Activation of microglia acidifies lysosomes and leads to degradation of Alzheimer amyloid fibrils. *Molecular Biology of the Cell*.

[99] Boissonneault V, Filali M, Lessard M, Relton J, Wong G, Rivest S (2009). Powerful beneficial effects of macrophage colony-stimulating factor on *β*-amyloid deposition and cognitive impairment in Alzheimer’s disease. *Brain*.

[100] Chihara T, Suzu S, Hassan R (2010). IL-34 and M-CSF share the receptor Fms but are not identical in biological activity and signal activation. *Cell Death and Differentiation*.

[101] Dalpke AH, Scháfer MKH, Frey M (2002). Immunostimulatory CpG-DNA activates murine microglia. *Journal of Immunology*.

[102] Iliev AI, Stringaris AK, Nau R, Neumann H (2004). Neuronal injury mediated via stimulation of microglial toll-like receptor-9 (TLR9). *The FASEB Journal*.

[103] Surh YJ, Kundu JK, Li MH, Na HK, Cha YN (2009). Role of Nrf2-mediated heme oxygenase-1 upregulation in adaptive survival response to nitrosative stress. *Archives of Pharmacal Research*.

[104] Kensler TW, Wakabayashi N, Biswal S (2007). Cell survival responses to environmental stresses via the Keap1-Nrf2-ARE pathway. *Annual Review of Pharmacology and Toxicology*.

[105] Kanninen K, Heikkinen R, Malm T (2009). Intrahippocampal injection of a lentiviral vector expressing Nrf2 improves spatial learning in a mouse model of Alzheimer’s disease. *Proceedings of the National Academy of Sciences of the United States of America*.

